# ‘apparent’: a simple and flexible R package for accurate SNP-based parentage analysis in the absence of guiding information

**DOI:** 10.1186/s12859-019-2662-3

**Published:** 2019-02-28

**Authors:** Arthur T. O. Melo, Iago Hale

**Affiliations:** Department of Agriculture, Nutrition, and Food Systems, University of New Hampshire, Durham, NH USA

**Keywords:** Parentage analysis, Pedigree inference, R package, SNP

## Abstract

**Background:**

The accurate determination of parent-progeny relationships within both in situ natural populations and ex situ genetic resource collections can greatly enhance plant breeding/domestication efforts and support plant genetic resource conservation strategies. Although a range of parentage analysis tools are available, none are designed to infer such relationships using genome-wide single nucleotide polymorphism (SNP) data in the complete absence of guiding information, such as generational groups, partial pedigrees, or genders. The R package (‘apparent’) developed and presented here addresses this gap.

**Results:**

‘apparent’ adopts a novel strategy of parentage analysis based on a test of genetic identity between a theoretically expected progeny (EP_ij_), whose genotypic state can be inferred at all homozygous loci for a pair of putative parents (*i* and *j*), and all potential offspring (PO_k_), represented by the *k* individuals of a given germplasm collection. Using the Gower Dissimilarity metric (GD), genetic identity between EP_ij_ and PO_k_ is taken as evidence that individuals *i* and *j* are the true parents of offspring *k*. Significance of a given triad (parental pair_ij_ + offspring_k_) is evaluated relative to the distribution of all GD_ij|k_ values for the population. With no guiding information provided, ‘apparent’ correctly identified the parental pairs of 15 lines of known pedigree within a test population of 77 accessions of *Actinidia arguta*, a performance unmatched by five other commonly used parentage analysis tools. In the case of an inconclusive triad analysis due to the absence of one parent from the test population, ‘apparent’ can perform a subsequent dyad analysis to identify a likely single parent for a given offspring. Average dyad analysis accuracy was 73.3% in the complete absence of pedigree information but increased to 100% when minimal generational information (adults vs. progeny) was provided.

**Conclusions:**

The ‘apparent’ R package is a fast and accurate parentage analysis tool that uses genome-wide SNP data to identify parent-progeny relationships within populations for which no a priori knowledge of family structure exists.

**Electronic supplementary material:**

The online version of this article (10.1186/s12859-019-2662-3) contains supplementary material, which is available to authorized users.

## Background

Parent-progeny relationships, whether among individuals within in situ natural populations or ex situ genetic resource collections, are of fundamental interest to plant and animal breeders, molecular ecologists, and population geneticists. As empirical records of gene flow, pedigrees provide insight into a species’ mating system [1], including patterns of compatibility within and among gene pools [2]. In plant improvement programs, pedigrees can directly inform breeding strategies [3, 4] by facilitating the estimation of breeding values [5, 6], heritabilities [7], and relative combining abilities [8, 9]. Knowledge of family structure can also help rationalize germplasm collections [10–12] and guide the management of natural resources [13–15], including strategies for reintroducing captive stock to their natural habitats [16, 17].

The basic theoretical principle underlying parentage analysis is that parent(s) can be assigned to their respective progeny with a certain level of confidence based on the signature of genetic compatibility between generations. In other words, Mendelian laws of inheritance permit the inference of genealogical relationships, provided one has a sufficiently informative set of genetic markers that stably transmits from parents to offspring [18]. Over the years, parentage analyses have used various classes of molecular markers for this purpose, including simple sequence repeats (SSRs), variable number tandem repeats (VNTRs), amplified fragment length polymorphisms (AFLPs), and restriction fragment length polymorphisms (RFLPs). Of these, SSRs have long been held as the most appropriate markers for such analyses due to their co-dominant nature, their high polymorphic content per locus, and their relative ease of scoring [19]. Recently, however, SSR genotyping has become less common, particularly in heretofore unstudied species, due to the comparative advantages of high-throughput, sequence-based genotyping methods.

High marker number and density, genome-wide coverage, ever falling cost per datapoint, and ongoing innovation in bioinformatic pipelines [20–25] have made sequence-based markers, particularly single nucleotide polymorphisms (SNPs), the current standard platform for genotyping in both model and non-model species [26]. The majority of available parentage analysis tools were originally developed for SSR data [13, 18], with an assumption of relatively small datasets (dozens to hundreds of data points). Although both SSRs and SNPs are co-dominant markers, such tools are unable to make efficient use of genome-wide SNP data (thousands to hundreds of thousands of data points). While some more recent parentage analysis algorithms have been developed to deal with such large datasets [27–30], all require some a priori knowledge of family structure for their implementation. That is, one must specify, at least, the basic generational structure (i.e. which lines are offspring and which are potential parents) up front in order to perform a robust parentage test. For species whose individuals are particularly long-lived (e.g. trees), difficult to age (e.g. woody lianas), or inbred long ago (e.g. many landraces of cereals), even such minimal information may be unavailable.

There is a rich history of developing relationship inference methods outside of the plant sciences, particularly in the context of both human and natural animal populations [13, 31–34]. Accurate knowledge of family structure among human subjects is critical to the unbiased assessment of linkage between genetic markers and diseases. Indeed, common relationship misclassifications due to false paternity assignments, unrecorded adoptions, or sample switches can lead to a loss of power in association studies [33, 35]. Several methods have been developed to address this issue; but it is worth noting that all are based on maximum likelihood and/or Bayesian approaches that require a priori knowledge of generational classifications, parental genders, putative pedigrees, family groups, and/or marker linkage [35, 36].

There remains, therefore, a need for a simple and robust parentage analysis tool that makes efficient use of large genomic datasets and requires no prior information about family structure. The ‘apparent’ package was developed with this need in mind; and below we describe its underlying strategy, compare its functionality and performance to existing tools, and report its availability.

## Implementation

### Description of strategy, use, and package availability

The ‘apparent’ analysis begins with a tab-delimited input table of SNP-based genotypes across some set of loci (columns) for all individuals (rows) in the target population (see Additional file 1). In column 2 of the input file, each individual in the population is assigned to one of five classes for the analysis: Mo (exclusively considered as a potential mother, or female parent), Fa (exclusively considered as a potential father, or male parent), Off (exclusively considered as an offspring), Pa (exclusively considered as a parent, both female and male), or All (considered as a potential female parent, male parent, and offspring within the population).

For each of the possible pairs of *i* female parents (Mo, Pa, and All) and *j* male parents (Fa, Pa, and All), the genotype of the Expected Progeny (EP_ij_) is constructed based only on markers that are homozygous in both parents. A rapid, pairwise calculation of genetic distance, namely Gower’s Dissimilarity coefficient (GD) [37], is then carried out between each EP_ij_ and all *k* potential offspring (PO_k_) in the population (Off and All). Ranging from 0 (perfect identity) to 1 (perfect dissimilarity), GD captures the degree of genetic relatedness between two individuals by quantifying the identity-by-state of all *n* SNPs, according to:

1 $$ {GD}_{ij\mid k}\left(\operatorname{}{EP}_{ij}|{PO}_k\right)=1-\left(\frac{\sum \limits_{l=1}^n{s}_l{w}_l}{\sum \limits_{l=1}^n{w}_l}\right) $$

where, for each SNP_l_, s_l_ = 1 if the genotypic states are the same; s_l_ = 0.5 if the genotypic states differ by one allele (i.e. heterozygote vs. homozygote); s_l_ = 0 if the genotypic states differ by both alleles (i.e. primary homozygote vs. secondary homozygote); w_l_ = 1 if both individuals are genotyped; and w_l_ = 0 if either individual lacks an assigned genotype (e.g. missing data due to low coverage).

**Fig. 1:**
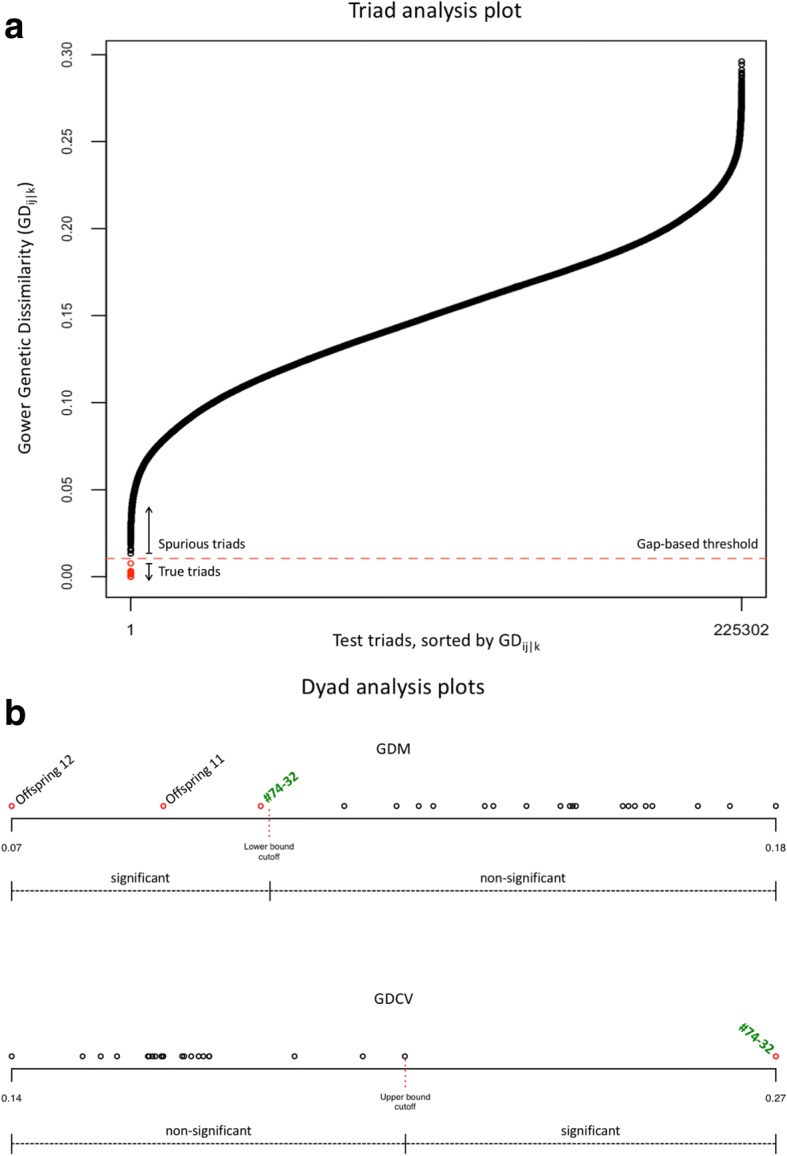
The ‘apparent’ analysis plots. For a given population, a simple gap analysis separates true triads from spurious relationships. (**a**) Gower Dissimilarities (GD_ij|k_) are plotted for all possible parent-offspring combinations in the population, enabling an inspection of gap size and all subsequent hypothesis testing. (**b**) For each significant parent-offspring association from the dyad analysis, distribution plots of mean GD_i(1...j)|k_ values (GDM) and their standard deviation in units of GD_i|k_ (GDCV) help visualize the analysis. In this particular example, *A. arguta* cv. ‘#74–32’ was correctly identified as a parent of offspring 10 despite the absence of the other parent (cv. ‘Chang Bai Mountain 5’) from the population and the confounding presence of two full-sibs (offspring 11 and 12)

Theoretically, if Mo_i_ and Fa_j_ are the true parents of PO_k_, EP_ij_ and PO_k_ will be genetically identical across all homozygous parental loci, resulting in a pairwise GD equal to zero. Due to both sequencing and genotyping errors, however, in practice the calculated GD value for a true triad (Mo_i_, Fa_j_, PO_k_) will be greater than zero; but it will be significantly lower than the population of GD’s calculated between EP_ij_ and all false offspring. Indeed, for a given population of individuals, a scatterplot of all possible GD_ij|k_ values exhibits a significant gap that separates true triads from spurious associations (Fig. 1a). This gap is located by scanning the ordered set of GD_ij|k_ values and detecting the place of maximum difference between two adjacent values; and the midpoint of this gap is taken as a simple threshold (Fig. 1a). A similar approach has been described as a reliable means of separating true and false parent-offspring assignments when applying discriminant analysis to thousands of homozygous loci [30, 38].

Once the gap has been identified, the significance of its magnitude vis-à-vis the distribution of gap lengths throughout the plot is assessed via a Dixon test [39, 40]. If the size of the gap is declared significant, the individual significance of each triad below the gap (i.e. those triads declared as potential real parent-offspring associations) is then tested against a sample of the most closely-related GD_ij|k_ values above the gap (i.e. those triads declared as spurious). If this second Dixon test is also found to be statistically significant, the implicated triad is declared as true and its *p*-value reported.

In the above triad analysis, a given offspring can be assigned to a pair of parents if and only if all three individuals (both parents and the offspring) are present in the genotyped population. In an attempt to identify one parent despite the absence of the other in the population, a subsequent dyad analysis can be performed. The primary challenge of such an analysis lies in discriminating an individual’s true parent from other close relatives (e.g. full siblings). To address this challenge, ‘apparent’ conducts a two-stage statistical test.

The first test makes use of the fact that, on average, an individual is more closely related to a population of its siblings than it is to a population of random individuals. For each potential offspring *k* and potential parent *i*, the package calculates the mean GD (GDM) between that PO_k_ and all expected progeny arising from the *j* possible triads involving potential parent *i*:

2 $$ GDM\equiv \frac{1}{j}{\sum}_j{GD}_{\left. ij\right|k} $$

For each PO_k_, the resulting set of GDM values, one for each parent *i*, is treated as a normal distribution and the normal score of each value is obtained. If any normal score falls below the lower bound of the user-defined confidence interval, the pair (parent *i* and PO_k_) is flagged as a potential parent-progeny set.

The second test makes use of the fact that, on average, variation in GD is higher between an individual and a population of its siblings than between an individual and a population of the progeny of its siblings. To further test the potential parent-progeny sets flagged above, the ‘apparent’ dyad analysis thus considers the variation within the sets of GD_i(1...j)|k_ values. Specifically, for each PO_k_ and potential parent *i*, the package calculates the standard deviation among the pairwise GD’s between PO_k_ and each expected progeny arising from the *j* triads involving potential parent *i*:

3 $$ {\sigma}_{GD_{\left.i\left(1\dots j\right)\right|k}}=\sqrt{\frac{1}{j-1}{\sum}_j{\left({GD}_{\left. ij\right|k}-\frac{1}{j}{\sum}_j{GD}_{\left. ij\right|k}\right)}^2} $$

For the purpose of testing against the background of the entire population, this standard deviation is re-expressed in units of GD_i|k_, the Gower Dissimilarity between PO_k_ and potential parent *i* itself:

4 $$ GDCV\equiv \frac{\sigma_{G{D}_{i\left(\operatorname{}1\dots j|\right)k}}}{G{D}_{i\mid k}} $$

Similar to the first test above, for each PO_k_ the resulting set of GDCV values, one for each parent *i*, is treated as a normal distribution and the normal score of each value is obtained. If any normal score exceeds the upper bound of the user-defined confidence interval, the pair (parent *i* and PO_k_) is reported as a likely potential parent-progeny set, along with its cumulative *p*-value. As shown in Fig. 1b, this two-step dyad analysis is effective not only in identifying likely parents (significant outliers in both tests) but also in distinguishing such parents from other close relatives (significant outliers in the first test only).

It is important to note that the ‘apparent’ algorithm makes no assumptions about the ploidy of the species under investigation; and the strategy performs well for any level of available pedigree information, from none (completely unknown adults and offspring) to the maximum possible information available (known adults, including their genders, as well as the set of offspring). The simple approach accommodates unlimited markers across unlimited individuals, the only requirement being that the population under investigation is genotyped with bi-allelic SNP markers. The ‘apparent’ package is freely available at https://github.com/halelab/apparent and through the Comprehensive R Archive Network (CRAN) at https://cran.r-project.org.

### Method validation

To test the validity of the approach described above, we turned to the North American kiwiberry (*Actinidia arguta*) collection, comprised of 62 tetraploid (2n = 4x = 116), dioecious genotypes [41]. From these 62 genotypes, four males and five females were used in controlled crosses to produce a total of 15 offspring of known parentage (five groups of three full-siblings each; see Additional files 2 and 3). For each of the 77 samples (62 + 15 offspring), genomic DNA was isolated from ~ 1 g of fresh young leaves using a modified CTAB protocol, cleaned with a spin column (Zymo Research, Genomic DNA Clean & Concentrator™-10), and multiplexed into genotyping-by-sequencing (GBS) libraries using a two enzyme (*PstI*-*MspI*) protocol [42]. The libraries were sequenced using 150 bp paired-end (PE) reads on an Illumina 2500 HiSeq platform, and the CASAVA-processed sequence data were submitted to the GBS-SNP-CROP pipeline [25] for genotyping. Stringent quality filtering was carried out, as explained in detail in the pipeline documentation; and all recommended ploidy-specific parameters were used for SNP calling and genotyping.

The resulting set of genotypic data was submitted to ‘apparent’ with no accompanying generational, gender, or pedigree information. In other words, all 77 genotypes were coded as ‘All’ in the input file, meaning each individual was to be considered by ‘apparent’ as a possible mother, father, and offspring, for a total of 225,302 potential triads. Package performance was assessed using the following four metrics: 1) Number of Type I errors (false triads declared true); 2) Number of Type II errors (undeclared true triads); 3) Overall accuracy [100 * Number of declared true triads/(Number of true triads + Number of false triads declared true)]; and 4) Computation time.

Using the same set of data, we investigated the impact of total marker number on performance. Finally, we compared the simple gap-based method of triad GD threshold determination with a more intensive approach involving computation of genetic dissimilarities among technical replicates (i.e. duplicated DNA samples isolated from three different genotypes, split between different library preparations, and sequenced on different Illumina lanes).

### Comparison to other parentage analysis tools

After choosing an appropriate number of loci to include in the analysis, we compared the performance of ‘apparent’ with five other parentage analysis tools, including four R packages (‘MasterBayes’ *MCMCped* function [27], ‘ParentOffspring’ [28], ‘Solomon’ [29], and ‘hsphase’ *pogc* function [30]) and the Windows-based program Cervus [43, 44], one of the most widely used software tools for parentage analysis. As described above for ‘apparent,’ we evaluated the performances of these tools using the test population of 77 *A. arguta* accessions. To fairly compare performance among tools, we applied the same criteria to all analyses, namely: 1) The same set of 1000 SNPs was used; 2) All 225,302 potential triads were tested (i.e. no information was provided in terms of classifying individuals as mothers, fathers, or offspring); and 3) Confidence level, when supported by a given tool, was set at 99% (α = 1%).

In addition, a more qualitative comparison of the tools was done based on their main features, ease of use, and available functions. The main features considered were marker type, parentage analysis method, number of genotype classes that must be declared, and operating system compatibility. Ease of use considers the relative level of difficulty in parameterizing the various tools, creating the needed input files, and interpreting the output. Lastly, the comparison of available functions follows the typology proposed by Jones et al. 2010 [18] to classify the various tools based on their abilities to perform paternity/maternity, parent pair allocation, parental reconstruction, sib-ship reconstruction, and full probability analyses. Also considered are the tools’ abilities to calculate exclusion probabilities, assign statistical confidence to individual parent-offspring pairs, and assess experiment-wide statistical confidence of parent-offspring assignments.

## Results and discussion

GBS-SNP-CROP retained, on average, 5.14 million high-quality PE reads per genotype (Additional file 2) and called a total of 27,852 SNPs, with an average depth D = 36.0. Overall levels of heterozygosity, homozygosity, and missing data were 36.6, 51.5, and 11.8%, respectively.

### Optimizing SNP number for parentage analysis

**Fig. 2:**
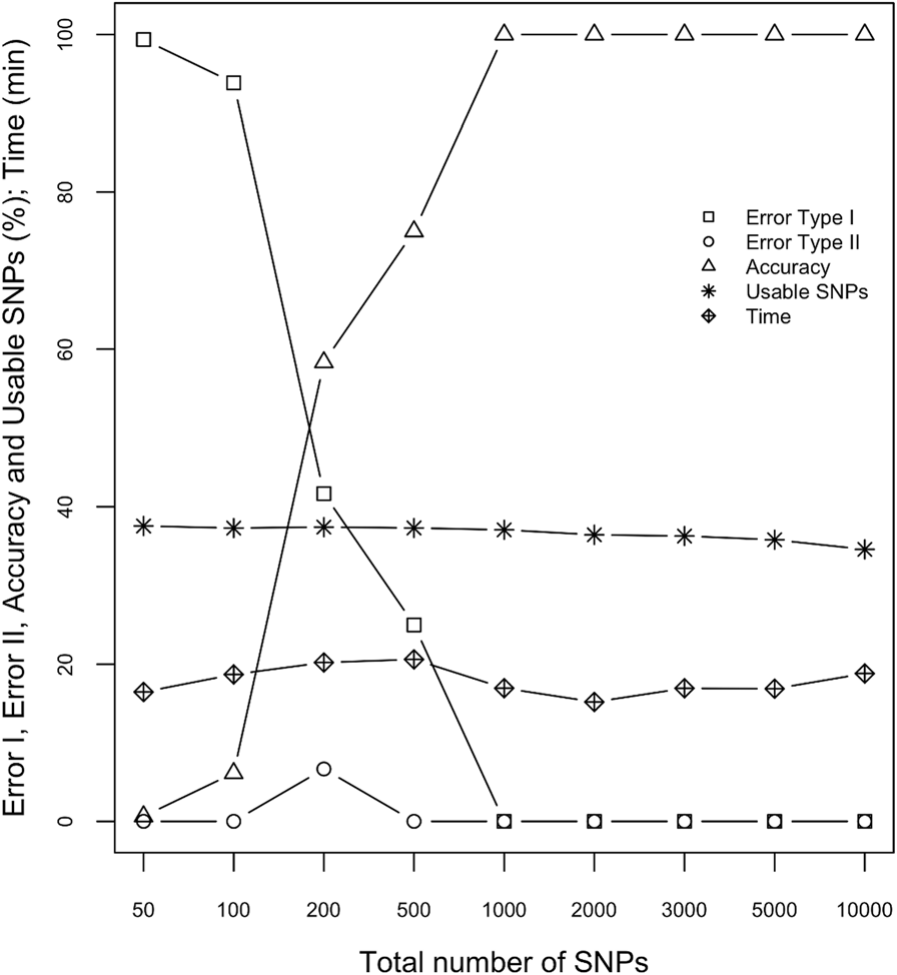
Influence of the number of SNP loci on error rates, accuracy, and computation time. For each set of loci sampled, the performance of the ‘apparent’ package was evaluated in terms of error rates (Types I and II) and accuracy. The times required to successfully complete the analyses were also recorded and reveal a surprising insensitivity to the number of markers used. Note that the percentage of markers usable by ‘apparent’ for the analysis (i.e. parental homozygous SNPs) is quite stable

From the 27,852 SNPs called, random subsets of various sizes, ranging from 50 to 10,000 SNPs, were sampled and evaluated. Because only pairwise homozygous loci are used by ‘apparent’ for analysis, the genotype of any given EP_ij_ is based on fewer SNPs than the total available. For example, when 50 SNPs were provided to ‘apparent’, only 19 were usable in the analysis of this population; and the result was both a very high Type I error rate (99.4%) and a very low overall accuracy (0.64%). Supplying 500 SNPs to the package increased the number of usable loci to 186, which decreased the Type I error rate substantially (25.0%) and greatly improved overall accuracy (75.0%). With 1000 loci (371 SNPs used), the model became stable with no errors (100% accuracy) (Fig. 2).

Although 1000 was found to be the lowest acceptable number of loci for reliable parentage analysis within this *A. arguta* collection, the optimum number can be expected to vary according to the species under investigation, the diversity within and among lines, and the population structure. For example, parentage analysis within a highly heterozygous, outcrossing species may require a relatively larger pool of loci due to the fact that a small proportion will be homozygous for any given pair of possible parents. In comparison, a greater proportion of loci generally will be usable in a more homozygous, inbred species, thereby requiring a relatively smaller pool of loci. In practice, as long as all of the individuals in the analysis can be clearly discriminated from one another based on the available pairwise homozygous loci, there will be sufficient resolution for the ‘apparent’ analysis. And as discussed in more detail below, increasing the number of loci has very little effect on total computation time; so there is no real advantage to using a reduced marker set.

### Accuracy and computation time

Using 1000 total SNPs, ‘apparent’ identified the parental pairs of all 15 offspring from the controlled crosses with 100% accuracy (no Type I or II errors), despite the complicating presence of full-sibs in the population. In addition, we found an average accuracy of 73.3% (range 33.3–100%) for dyad analysis, over the nine analyses where one male or one female parent of the known offspring was removed from the population. Dyad analysis reached a consistent 100% accuracy, however, when minimal generational information (adults vs. juveniles) was provided to the algorithm. Both the triad and dyad analyses produce easily parsable and tab-delimited output (Additional file 4), along with summary plots (Fig. 1).

While the pairwise GD between redundant genotypes (i.e. technical replicates) should in theory be zero, the existence of both sequencing and genotyping errors means that, in practice, perfect similarity is rarely observed. Using the summary plot of GD_ij|k_ values, ‘apparent’ adopts a simple gap-based method of GD threshold determination to separate putative true triads from spurious parent-progeny associations. For the test population of 77 *A. arguta* accessions, the true triads identified via the gap-based method had a mean GD_ij|k_ of 0.0016. In a previous study with this population [35], 99% confidence intervals for declaring redundancy were empirically determined based on distributions of GD’s obtained between pairs of both biological replicates (two independent DNA isolations from the same accession, prepared as part of the same GBS library and sequenced in the same lane) and technical replicates (a single DNA isolation, used in two separate GBS library preparations and sequenced on different lanes). The mean GD_ij|k_ for triads declared via the gap-based method is lower than both the biological (0.0024) and technical (0.0046) replicate thresholds, meaning the simple gap-based ‘apparent’ assignments are supported by empirical measures of genetic redundancy.

Recognizing that true triads exhibit a very small pairwise GD_ij|k_, despite the presence of sequencing and genotyping errors, one can greatly accelerate the ‘apparent’ analysis by limiting the time-intensive gap analysis to only those GD_ij|k_ values below some user-specified threshold via the package’s MaxIdent parameter. The MaxIdent default of 10% greatly reduces the analysis time because all GD_ij|k_ values above 0.1 are ignored during significance testing (i.e. they cannot, by definition, be declared as true triads). In a test population of *n* = 77 individuals, each coded as ‘All’ (potential mothers, fathers, and offspring), pairwise GD_ij|k_ values for a total of 225,302 possible triads must be explored [n^2^ * (n-1)/2]. With MaxIdent set to 0.1, however, the computation time required by ‘apparent’ for the *A. arguta* test population is modest (~ 20 min on a Unix workstation with a 2.6 GHz Dual Intel processor and 16 GB RAM) and fairly insensitive to the number of loci used (Fig. 2).

As a final note on computation time, although increasing the number of loci for a given population has very little effect on total computation time, increasing the number of individuals in that population does. In the absence of guiding information (i.e. all individuals coded as ‘All’), the exploratory triad space grows as the cube of the population size, an inflation that directly influences required computation time (see Additional file 5). Users are therefore advised to minimize the size of the exploratory triad space on the basis of available gender and/or generational information. Indeed, excluding irrelevant triads from the analysis should be considered a best practice, along with including a known triad in the population (i.e. a control) and culling individuals with unusually low mean GD_ij|k_ values or mean usable number of loci (see https://github.com/halelab/apparent for details).

### Comparing features and performance with other tools

As summarized in Table 1, the ‘apparent’ package offers a novel combination of features compared to those possessed by the following commonly used parentage analysis tools: ‘MasterBayes’ *MCMCped* function [27], ‘ParentOffspring’ [28], ‘Solomon’ [29], ‘hsphase’ *pogc* function [30], and Cervus [43, 44]. Only ‘apparent’ and ‘hsphase’ permit fully exploratory parentage analysis in the absence of a priori classifications of individuals (e.g. parents vs. offspring). Despite this point of commonality, ‘apparent’ greatly exceeds the functionality of ‘hsphase’ in its performance of both paternity/maternity analysis and parent pair allocation, not to mention its ability to assign statistical confidence to declared triads. The ‘apparent’ package was also designed with relative ease of use in mind, a result accomplished via simple parameterization, input file requirements, and output interpretation.

**Table 1:** Comparison of the ‘apparent’ R package to five currently available tools for parentage analysis, based on main features, ease of use, and available functions

Package/Tool	Main features				Ease of use			Available functions^e^							
	Marker Type^a^	Method of Analysis^b^	Required classes^c^	OS^d^	Parameter-ization	Input	Output	PM	PP	PR	SR	FP	EP	IC	EC
Apparent	Co	GR/GS	none	R	Easy	Easy	Easy	X	X	X				X	
hsphase (*pogc*)	Co	GR/GS	none	R	Easy	Easy	Complex			X	X				
MasterBayes (*MCMCped*)	Co/Do	BA/ML	Pa/Off	R	Complex	Easy	Easy	X	X	X		X		X	X
ParentOffspring	Co	GR/GS	Mo/Fa/Off	R	Easy	Easy	Easy		X						
Solomon	Co	BA	Pa/Off	R	Moderate	Easy	Moderate	X	X				X	X	X
Cervus	Co/Do	ML	Pa/Off	W	Moderate	Easy	Moderate	X	X				X	X	X

^a^*Co* = Co-dominant, *Do* = Dominant

^b^*BA* = Bayesian approach; *GR* = Genetic relatedness, *GS* = Genetic similarity, *ML* = Maximum likelihood

^c^*none* = no a priori information required for individuals, *Pa/Off* = each individual must be classified as either a Parent or an Offspring [2 classes], *Fa/Mo/Off* = each individual must be uniquely classified as a potential Mother, Father, or Offspring [3 classes]

^d^Operational System. *R* = R package (OS independent), *W* = Windows

^e^Following the typology developed by Jones et al. (2010): *PM* = Paternity/maternity analysis, *PP* = Parent pair allocation, *PR* = Parental reconstruction analysis, *SR* = Sib-ship Reconstruction, *FP* = Full probability analysis, *EP* = Ability to calculate exclusion probabilities, *IC* = Ability to assign statistical confidence to individual parent-offspring pairs, *EC* = Ability to assess experiment-wide statistical confidence of parent-offspring assignments

In addition to occupying a unique niche among available parental analysis tools in terms of features, ‘apparent’ consistently outperformed those tools in the correct identification of parent-offspring triads in the test population of 77 *A. arguta* individuals. Applying the same criteria to all analyses, the overall accuracy of the five tools ranged from 2.3–55.6%, compared to 100% for ‘apparent’ (Table 2). Cervus, one of the most popular parentage analysis tools available, completed the analysis in just under 12 min with no Type II errors; but it committed 44 Type I errors out of a total of 59 declared significant triads. Despite these errors, Cervus proved to be one of the better overall tools of the five, with an accuracy of 50.8%. These results indicate that identifying correct parent-offspring assignments within a population lacking pedigree information is a challenge even for one of the most robust parentage analysis tools available. Notably, Cervus’ triad accuracy increased to 100% when generational information (i.e. which individuals are parents and which are offspring) was supplied to the algorithm (Table 2).

**Table 2:** Summary of results comparing the performance of ‘apparent’ to five other parentage analysis tools in identifying the pairs of parents of 15 *A. arguta* offspring in a population of 77 individuals

Package/Tool	Analysis with no guiding information provided				Analysis guided with 2 genotypic classes: adults vs. offspring				Citation
	Error I (%)	Error II (%)	Accuracy (%)	Time (min)	Error I (%)	Error II (%)	Accuracy (%)	Time (min)	
apparent	0.0	0.0	100.0	18.4	0.0	0.0	100.0	3.1	–
hsphase (*pogc*)	31.0	5.7	17.4	0.1	31.0	5.7	17.4	0.1	Ferdosi et al. (2014) [30]
MasterBayes (*MCMCped*)	15.6	22.1	48.2	2.3	0.0	0.0	100.0	0.3	Hadfield et al. (2006) [27]
ParentOffspring^a^	44.4	0.0	55.6	260.4	6.3	0.0	93.8	28.9	Abdel-Haleem et al. (2013) [28]
Solomon	97.7	0.9	2.3	401.4	97.5	1.0	2.6	43.1	Christie et al. (2013) [29]
Cervus	74.6	0.0	50.9	11.9	0.0	0.0	100.0	5.3	Kalinowski et al. (2007) [44]

^a^The results from ParentOffspring are approximate because the package requires an a priori declaration of parental pairs for a given set of offspring (see main text)

In the absence of a priori classifying information, ‘MasterBayes’ and ‘ParentOffspring’ exhibited similar overall accuracies (48.1 and 55.5%, respectively; Table 2). The categorical allocation analysis of ‘MasterBayes’ relies on a Markov Chain Monte Carlo approach and runs extremely fast (Table 2); and the package is arguably one of the most sophisticated and comprehensive parentage analysis tools available, owing to its ability to handle both co-dominant and dominant markers and to perform Full Probability analysis (Table 1). The low accuracy of ‘MasterBayes’ in this scenario is understandable, however, in light of the fact that its modeling framework lies firmly within the tradition of analyses developed for general, guided relationship inference in human populations [35, 36], as opposed to the single, well-defined task of unguided parent identification under consideration here. As with Cervus, the accuracy improves greatly (100%) when generational classifications (parents vs. offspring) are provided. Unlike Cervus, however, ‘MasterBayes’ is noteworthy in its difficulty of use, a result of its complex input file requirements and non-trivial parameterization.

To run the ‘ParentOffspring’ package, generational classifications (parents vs. offspring) are required; therefore, carrying out a full, unbiased exploration of the full triad space (225,302 triads) is extremely cumbersome. Even when the required generational classifications (i.e. designating the 15 known offspring as juveniles) were provided, however, the algorithm committed one Type I error (Table 2). Reducing the guiding information even a little, by classifying some full-sib offspring as adults and adults of the same gender as potential parental pairs, increased the number of Type I error significantly and decreased the model accuracy to 55.5%. Given the impracticality of manually running all combinations of the 77 genotypes, the computation time to complete the whole analysis was estimated to be ~ 261 min, not including the time required for the manual permutation of the input files.

The ‘hsphase’ parentage assignment function *pogc* was only 26.1% accurate in this scenario of no available pedigree information. This was a somewhat surprising result, given the fact that both ‘hsphase’ and ‘apparent’ exclusively use homozygous parental loci for discriminating true and false parent-offspring assignments. Unlike ‘hsphase’, however, the ‘apparent’ GD_ij|k_ gap value is extensively tested based on outlier prediction (Dixon test), allowing the inference of statistical confidence for declared triads.

Of all the packages tested, ‘Solomon’ showed the worst overall performance, with an accuracy of only 2.3% in this scenario of no available pedigree information. In addition, the computational time required by ‘Solomon’ to complete the analysis was significantly longer than all other packages (401 min) due to the fundamental dependencies inherent in Bayesian approaches. Surprisingly, the package’s accuracy rose to a mere 2.6% when the adults and the offspring were duly classified; and in both scenarios the Type I error rate was around 97% (Table 2).

Compared to other available tools, the simplicity, speed, and accuracy of the ‘apparent’ package recommend it as a useful tool for inferring parent-offspring relationships within populations for which a priori relational information is lacking. The key column of the simple input file (Additional file 1, second column) lies at the heart of the package’s flexibility, allowing individuals in the population to be tested as both parents and offspring in the same analysis and eliminating the requirement for pedigree information. This same column also allows the user to provide additional information if it is available; thus one can easily control the type of parentage analysis performed. For example, if generational information (adults vs. offspring) and adult genders are known, either paternity or maternity analyses can be performed. If the genders are unknown, a generation-guided categorical allocation analysis is performed. Finally, when no family information is available and all individuals are to be tested as potential mothers, fathers, and offspring, ‘apparent’s novel approach to unguided categorical allocation is carried out, filling a current gap among existing parentage analysis tools.

## Conclusions

By offering quick and accurate inference of parent-offspring triads within populations for which no generational, gender, or pedigree information is available, the ‘apparent’ R package occupies a unique niche among currently available parentage analysis tools. With simple parameterization and easily interpretable output, the package should be considered by molecular ecologists, population geneticists, and breeders interested in evaluating family relationships within populations of either model and non-model species for which genome-wide SNP data are available.

In terms of its range of applicability, it is worth emphasizing the fact that ‘apparent’ only attempts to identify *direct* parent-offspring associations (i.e. the approach only looks back a single generation to identify immediate parents). In practice, then, unless every line from all stages of a breeding program is genotyped (highly unlikely for annual crops), the required genomic data will not be available to establish the chain of generations underlying certain pedigrees of interest (e.g. the original parents of an inbred line). For this reason, the approach is more practically suited to questions of direct parentage within long-lived species, for which multiple generations co-exist and can therefore be included together in the analysis (e.g. trees, woody lianas, other perennials, clonally-propagated crops, etc.). In other words, ‘apparent’ is arguably best suited to plant species which cohere to the animal model, in the sense of having co-existing parents and offspring.

## Availability and requirements

**Project name:** apparent.

**Project home page:** https://github.com/halelab/apparent.

**Operating system(s):** Platform independent.

**Programming language:** R.

**Other requirements:** R (> = 3.0.2).

**License:** GPL (> = 2).

**Any restrictions to use by non-academics:** none.

## Additional files

Additional file 1: An example input file for the ‘apparent’ package, featuring a population of 20 individuals, genotyped at ten SNP loci. Column one of the input file contains the ID’s of the individuals in the population. Column two contains a classification key which assigns each individual to one of five possible classes for analysis: Mo = potential mother; Fa = potential father; Pa = potential parent (either Fa or Ma); Off = potential offspring; and All = all possible classes (i.e. there is no previous information, and the individual will be tested as a potential Fa, Ma, and Off). The third and all subsequent columns contain genotype calls, with one SNP per column and the alleles separated by “/”. A missing genotype is represented as “−/−”. The tab-delimited input file has no column headers. (TXT 1 kb)

Additional file 2: Details of the population of 77 *Actinidia arguta* individuals used in this study. For each accession, the following information is provided: Genotype name; Genotypic class, if part of a controlled cross for this study (Fa = Father; Ma = Mother; Off = Offspring); USDA Plant Introduction (PI) number(s), if assigned; USDA Corvallis *Actinidia* (CACT) accession number, if assigned; USDA Davis *Actinidia* (DACT) accession number(s), if assigned; University of New Hampshire (UNH) ID, if assigned; Gender, if known; GBS library membership (1 to 4); GBS barcode assignment; Number of high-quality paired-end (PE) reads used for SNP calling; and The NCBI Short Read Archive (SRA) number. (XLSX 19 kb)

Additional file 3: Pedigrees of the 15 *Actinidia arguta* offspring used to assess the performance of parentage analysis tools in this study. (XLSX 10 kb)

Additional file 4: Examples of the three different output files produced by the ‘apparent’ package. *Worksheet 1*: apparent-Triad-All, a complete sorted table of all 225,302 triads considered in the *A. arguta* example (only the first and last 10 rows are shown, to reduce file size). *Worksheet 2*: apparent-Triad-Sig, a reduced list reporting only those triads deemed true, based on the user-defined significance level (e.g. 99%). *Worksheet 3*: apparent-Dyad-Sig, the statistically significant results of the dyad analysis when the female parent of Family 1 (cv. ‘Dumbarton Oaks’) was removed from the population. (XLSX 51 kb)

Additional file 5: Effect of population size on computation time, for a set of 1000 SNPs. In the absence of guiding information (i.e. all individuals coded as ‘All’), the exploratory triad space grows as the cube of the population size, an inflation reflected in the required computation time. (TIF 205 kb)

## Abbreviations

AFLP: Amplified fragment length polymorphism
bp: Base pair
D: Average read depth
EP_ij_: Theoretically expected progeny of parents *i* and *j*
GBS: Genotyping-by-sequencing
GD: Gower Dissimilarity metric
GDCV: The standard deviation among the pairwise GD’s between PO_k_ and each expected progeny arising from the *j* triads involving potential parent *i*, expressed in units of GD_i|k_
GD_ij|k_: GD between EP_ij_ and PO_k_, based on the homozygous loci in parents *i* and *j*
GDM: The mean GD between a given PO_k_ and all expected progeny arising from the *j* possible triads involving potential parent *i*
PE: Paired-end
PO_k_: Potential offspring *k* in the study population
RFLP: Restriction fragment length polymorphism
SNP: Single nucleotide polymorphism
SSR: Simple sequence repeat
VNTR: Variable number tandem repeat

## References

[1] Vandepitte K, Meyer TD, Jacquemyn H, Ruiz IR, Honnay O (2013). The impact of extensive clonal growth on fine-scale mating patterns: a full paternity analysis of a lily-of-the-valley population (*Convallaria majalis*). Ann Bot.

[2] Zheng X, Li L, Liang F, Tan C, Tang S, Yu S (2017). Pedigree-based genome re-sequencing reveals genetic variation patterns of elite backbone varieties during modern rice improvement. Sci Rep.

[3] Wang XR, Torimaru T, Lindgren D, Fries A (2010). Marker-based parentage analysis facilitates low input ‘breeding without breeding’ strategies for forest trees. Tree Genet Genomes.

[4] Shimono A, Wang XR, Torimaru T, Lindgren D, Bo K (2011). Spatial variation in local pollen flow and mating success in a *Picea abies* clone archive and their implications for a novel “breeding without breeding” strategy. Tree Genet Genomes.

[5] Blonk RJW, Komen H, Kamstra A, van Arendonk JAM (2010). Estimating breeding values with molecular relatedness and reconstructed pedigrees in natural mating populations of common sole, *Solea solea*. Genetics.

[6] Roos APW, Schrooten C, Druet T (2011). Genomic breeding value estimation using genetic markers, inferred ancestral haplotypes and the genomic relationship matrix. J Dairy Sci.

[7] Ritland K (2000). Marker-inferred relatedness as a tool for detecting heritability in nature. Mol Ecol.

[8] Chigeza G, Mashingaidze K, Shanaham P (2014). Advanced cycle pedigree breeding in sunflower. II: combining ability for oil yield and its components. Euphytica.

[9] Fan XM, Zhang YD, Jeffers DP, Bi YQ, Kang MS, Yin XF (2018). Combining ability of yellow lines derived from CIMMYT populations for use in subtropical and tropical mid-altitude maize production environments. Crop Sci.

[10] Staraz MDV, Bandinelli R, Boselli M (2007). This P, Boursiquot JM, Laucou V, Lacombe T, Vares D. Genetic structuring and parentage analysis for evolutionary studies in grapevine: kin group and origin of the cultivar sangiovese revealed. J. Amer. Soc. Hort. Sci..

[11] Rosyara UR, Sebolt AM, Peace C, Iezzoni AF (2014). Identification of the paternal parent of ‘Bing’ sweet cherry and confirmation of descendants using single nucleotide polymorphism markers. J Amer Soc Hort Sci.

[12] Urrestarazu J, Denancé C, Ravon E, Guyader A, Guisnel R, Feugey L (2016). Analysis of the genetic diversity and structure across a wide range of germplasm reveals prominent gene flow in apple at the European level. BMC Plant Biol.

[13] Jones AG, Ardren WR (2003). Methods of parentage analysis in natural populations. Mol Ecol.

[14] Garcia C, Jordano P, Godoy JA (2007). Contemporary pollen and seed dispersal in a *Prunus mahaleb* population: patterns in distance and direction. Mol Ecol.

[15] Guerier AS, Bishop JM, Crawford SJ, Küntzel AS, Stratford KJ. Parentage analysis in a managed free ranging population of southern white rhinoceros: genetic diversity, pedigrees and management. Conserv Genet. 2007. 10.1007/s10592-012-0331-4.

[16] Christie MR, Marine ML, French RA, Blouin MS (2012). Genetic adaptation to captivity can occur in a single generation. P Natl Acad Sci USA.

[17] Edwards T, Cox EC, Buzzard V, Wiese C, Hillard LS, Murphy RW. Genetic assessments and parentage analysis of captive bolson tortoises (*Gopherus flavomarginatus*) inform their “rewilding” in New Mexico. PLoS One. 2014. 10.1371/journal.pone.0102787. 10.1371/journal.pone.0102787 PMC4100913 25029369

[18] Jones AG, Small CM, Paczolt KA, Ratterman NL (2010). A practical guide to methods of parentage analysis. Mol Ecol.

[19] Pemberton JM (2009). Wild pedigrees: the way forward. P Roy Soc Lond B.

[20] Bradbury PJ, Zhang Z, Kroon DE, Casstevens TM, Ramdoss Y, Buckler ES (2007). TASSEL: software for association mapping of complex traits in diverse samples. Bioinformatics.

[21] Glaubitz JC, Casstevens TM, Lu F, Harriman J, Elshire RJ, Sun Q, Buckler ES (2014). TASSEL-GBS: a high capacity genotyping by sequencing analysis pipeline. PLoS One.

[22] Lu F, Lipka AE, Glaubitz J, Elshire RJ, Cherney JH, Casler MD (2013). Switchgrass genomic diversity, ploidy, and evolution: novel insights from a network-based SNP discovery protocol. PLoS Genet.

[23] Catchen JM, Amores A, Hohenlohe P, Cresko W, Postlethwait JH (2011). Stacks: building and genotyping loci de novo from short-read sequences. G3.

[24] Tinker NA, Bekele WA (2016). Hattori. Haplotag: software for haplotype- based genotyping-by-sequencing analysis. G3..

[25] Melo ATO, Bartaula R, Hale I (2016). GBS-SNP-CROP: a reference-optional pipeline for SNP discovery and plant germplasm characterization using variable length, paired-end genotyping-by-sequencing data. BMC Bioinformatics.

[26] Glaubitz JC, Rhodes E, Dewoody A (2003). Prospect for inferring pairwise relationship with single nucleotide polymorphisms. Mol Ecol.

[27] Hadfield JD, Richardson DS, Burke T (2006). Towards unbiased parentage assignment: combining genetic, behavioural and spatial data in a Bayesian framework. Mol Ecol.

[28] Abdel-Haleem H, Ji P, Boerma HR, Li Z. An R package for SNP marker-based parent-offspring tests. Plant Methods. 2013. 10.1186/1746-4811-9-44. 10.1186/1746-4811-9-44 PMC3842643 24245988

[29] Christie MR, Tennessen JA, Blouin MS (2013). Bayesian parentage analysis with systematic accountability of genotyping error, missing data, and false matching. Bioinformatics.

[30] Ferdosi MH, Kinghorn BP, van der Werf JHJ, Lee SH, Gondro C. Hsphase: an R package for pedigree reconstruction, detection of recombination events, phasing and imputation of half-sib family groups. BMC Bioinformatics. 2014. 10.1186/1471-2105-15-172. 10.1186/1471-2105-15-172 PMC4069276 24906803

[31] Walling CA, Pemberton JM, Hadfield JD, Kruuk LEB (2010). Comparing parentage inference software: reanalysis of a red deer pedigree. Mol Ecol.

[32] Harrison HB, Saenz-Agudelo P, Planes S, Jones GP, Berumen ML (2013). Relative accuracy of three common methods of parentage analysis in natural populations. Mol Ecol.

[33] Goring HH, Ott J (1997). Relationship estimation in affected sib pair analysis of late-onset diseases. Eur J Hum Genet.

[34] Min Y, Wenquan C, Yaning Y (2012). Correcting for biases in affected sib-pair linkage analysis caused by uncertainty in sibling relationship. SCIENCE CHINA Math.

[35] Epstein MP, Duren WL, Boehnke M (2000). Improved inference of relationship for pairs of individuals. Am J Hum Genet.

[36] Olson JM (1999). Relationship estimation by Markov-process models in a sib-pair linkage study. Am J Hum Genet.

[37] Gower JC (1971). A general coefficient of similarity and some of its properties. Biometrics.

[38] Calus MPL, Mulder HA, Bastiaansen JWM. Identification of Mendelian inconsistencies between SNP and pedigree information of sibs. Genet Sel Evol. 2011. 10.1186/1297-9686-43-34. 10.1186/1297-9686-43-34 PMC3377921 21988752

[39] Dixon WJ (1950). Analysis of extreme values. Ann Math Stat.

[40] Dixon WJ (1951). Ratios involving extreme values. Ann Math Stat.

[41] Melo ATO, Guthrie RS, Hale I. GBS-based deconvolution of the surviving north American collection of cold-hardy kiwifruit (*Actinidia* spp.) germplasm. PLoS One. 2017. 10.1371/journal.pone.0170580. 10.1371/journal.pone.0170580 PMC5268759 28125645

[42] Poland JA, Brown PJ, Sorrells ME, Jannink JL. Development of high-density genetic maps for barley and wheat using a novel two-enzyme genotyping-by-sequencing approach. PLoS One. 2012. 10.1371/journal.pone.0032253. 10.1371/journal.pone.0032253 PMC3289635 22389690

[43] Marshall TC, Slate J, Kruuk LEB, Pemberton JM (1998). Statistical confidence for likelihood-based paternity inference in natural populations. Mol Ecol.

[44] Kalinowski ST, Taper ML, Marshall TC (2007). Revising how the computer program CERVUS accommodates genotyping error increases success in paternity assignment. Mol Ecol.

