# Supplementary figures and images for: ‘apparent’: a simple and flexible R package for accurate SNP-based parentage analysis in the absence of guiding information
Source: BMC Bioinformatics. 2019 Feb 28;20:108. doi: 10.1186/s12859-019-2662-3 (PMC6396488; doi:10.1186/s12859-019-2662-3)

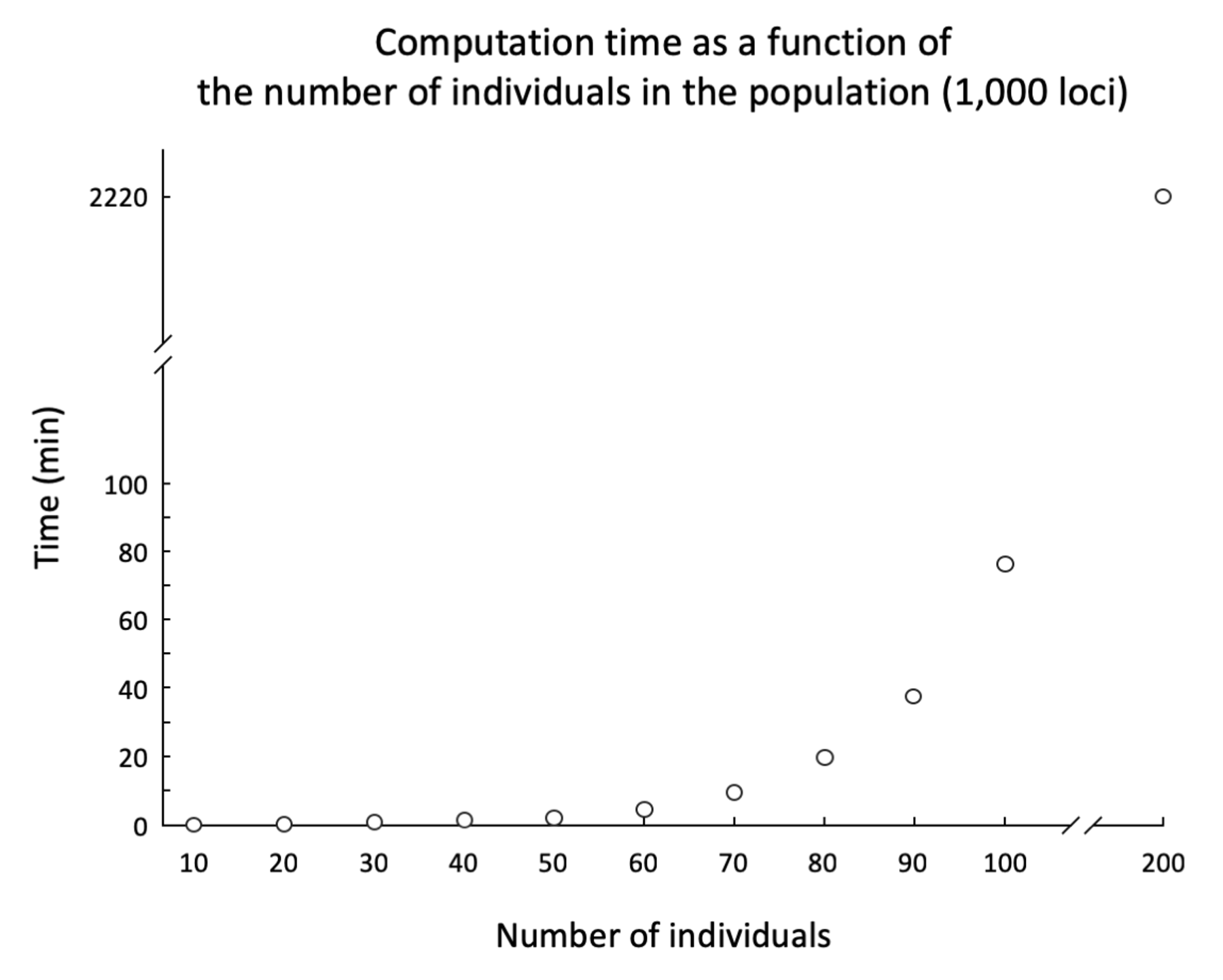

Supplement: Supplementary file 5 — Effect of population size on computation time, for a set of 1000 SNPs. In the absence of guiding information (i.e. all individuals coded as ‘All’), the exploratory triad space grows as the cube of the population size, an inflation reflected in the required computation time. (TIF 205 kb) [file 12859_2019_2662_MOESM5_ESM.tif]
